# *N-acetyltransferase 8*, a positional candidate for blood pressure and renal regulation: resequencing, association and *in silico *study

**DOI:** 10.1186/1471-2350-9-25

**Published:** 2008-04-10

**Authors:** Peeter Juhanson, Katrin Kepp, Elin Org, Gudrun Veldre, Piret Kelgo, Mai Rosenberg, Margus Viigimaa, Maris Laan

**Affiliations:** 1 Department of Biotechnology, Institute of Molecular and Cell Biology, University of Tartu, Tartu, Estonia; 2 Department of Cardiology, University of Tartu, Tartu, Estonia; 3 Department of Internal Medicine, University of Tartu, Tartu, Estonia; 4 Centre of Cardiology, North Estonia Medical Centre, Tallinn, Estonia

## Abstract

**Background:**

Kidneys have an important function in blood pressure (BP) regulation and elevated BP may lead to kidney failure. Chr2p12-p13 region linked to BP traits in multiple studies harbours a potential candidate for BP and renal function, *N-acetyltransferase 8 (NAT8) *expressed in embryonic and adult kidney and associated with nephrotoxicity response.

**Methods/Results:**

We report the first study exploring *NAT8 *as a potential candidate gene for blood pressure and kidney function. The resequencing (n = 42, random Estonian samples) identified 15 *NAT8 *polymorphisms, including 6 novel variants. The diversity of *NAT8 *5' upstream region (π/bp = 0.00320) exceeded up to 10 times the variation in the *NAT8 *genic region (π/bp = 0.00037) as well as the average variation (π/bp = 0.00040) for the promoters of 29 reference genes associated with hypertension. We suggest that a potential source for such high variation could be an active gene conversion process from *NAT8B *duplicate gene to *NAT8*. Similarly to *NAT8*, several reference genes with the most variable upstream regions have also duplicate copies. The *NAT8 *promoter SNPs were targeted with pilot quantitative association studies for blood pressure (n = 137, healthy unrelated individuals) and for the index of kidney function – estimated glomerular filtration rate (eGFR; n = 157 hypertensives with and without nephropathy). Minor alleles of these polymorphisms revealed a significant protective effect against elevated systolic BP as well as kidney failure in hypertension patients (p < 0.05; linear regression model, addictive effect).

**Conclusion:**

The full resequencing and pilot association study of a novel positional candidate gene for blood pressure and renal function, human *N-acetyltransferase 8*, suggested a contribution of highly variable *NAT8 *promoter polymorphisms in determination of systolic blood pressure and eGFR. Based on *in silico *analysis, we raise the hypothesis that the alternative SNP alleles of the *NAT8 *upstream region may have differential effect on gene expression.

## Background

Twin studies have shown that approximately half of the inter-individual variance in blood pressure (BP) level is heritable [1,2]. As distinctive for complex diseases, pathologically high BP diagnosed as essential hypertension (repeated systolic and diastolic BP readings > 140 and/or > 90 mmHg) evolves in association with genetic predisposition and personal life-style. Identification of the mutations behind Mendelian forms leading to hypo- or hypertension has highlighted the central role of the renal metabolic pathways in blood pressure regulation [3]. Consistently, depending on the severity and duration of the hypertension, the patients may develop morphological changes in vasculature of kidneys followed by the progression renal failure [4-6]. In the other way around, it has also been proposed that the defective embryonic development of the kidney or loss of existing nephrons may act as a predisposing factor for primary hypertension [7-10].

Genome-wide linkage studies [11] have identified the short-arm of human Chr2 as one of the four major genomic regions to be responsible for BP regulation and therefore hold a risk for essential hypertension. Several candidate genes (*ADD2; SLC8A1*) from this region have been proposed and investigated in detail to play a role in the aetiology of essential hypertension [12,13]. For this study we screened the reported linkage regions at Chr2 for novel BP regulating genes involved in kidney function and renal metabolic pathways. Within the window of 10 Mb from the four reported linkage peaks [11] we have identified at 2p13.1-p12 a novel functionally relevant candidate gene *N-acetyltransferase 8 (NAT8) *that is highly expressed in normal human embryonic kidney and liver. NAT8 protein has been suggested to play an important role in development and maintenance of the structure and function in adult kidney and liver [14]. Although exact function of the NAT8 is unknown, its expression profile is related to detoxification pathways in kidney [15].

In the present study, we have carried out the resequencing of human *NAT8 *gene followed by a pilot association study of the common *NAT8 *sequence variation with blood pressure measurements and the index of kidney function. We show that most of the *NAT8 *variation is harboured in the 5'UTR and upstream regions, which is associated with both, systolic blood pressure levels and the estimated glomerular filtration rate (eGFR).

## Methods

### Study Population

The study has been approved by the Ethics Committee on Human Research of University of Tartu, Estonia (permissions no 122/13,22.12.2003; 137/20, 25.04.2005). The initial study for resequencing (n = 42) and the association studies (detailed description found below) were carried out using samples of recruited individuals from the Estonian population, classified as a typical North-European population based on its genetic composition [16]. All participating individuals gave their informed consent prior to their inclusion into the study.

### Resequencing

**Figure 1:**
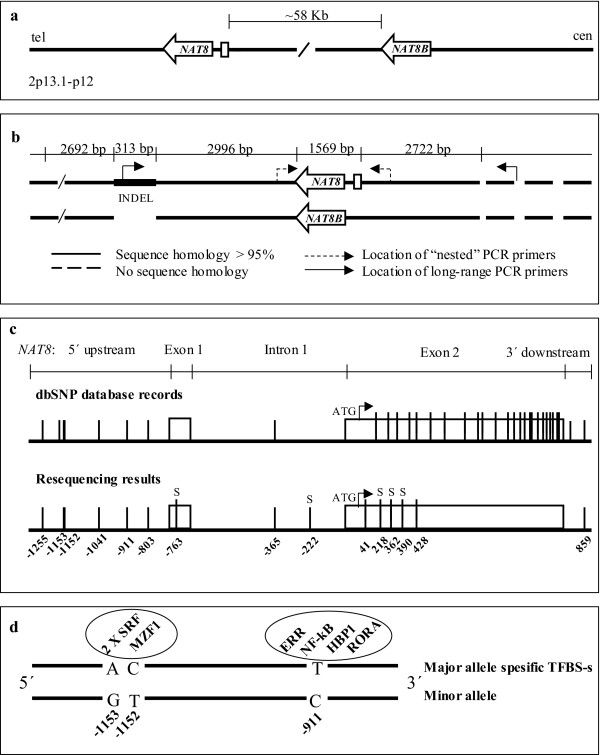
**(a) The structure of *NAT8/NAT8B *cluster: *NAT8*/*NAT8B *location in chromosome 2p13.1-p12; (b) Schematic presentation of the alignment of *NAT8*/*NAT8B *and their flanking regions and the positioning of *NAT8*-specific PCR primers; (c) *NAT8 *resequencing results and SNP positions compared to dbSNP database records; (d) Missing Binding Sites for Transcription Factors (TFBS) on chromosomes carrying the minor alleles of SNPs -911, -1151 and -1153.** "S" – singleton polymorphisms.

The sequence of the human *NAT8 *genomic region has been obtained from NCBI GeneBank database. As *NAT8 *possesses a duplicate gene *NAT8B *located about 58 Kb downstream from *NAT8*, the design of the amplification of human *NAT8 *gene involved two steps (long-range PCR and nested-PCR) (Figure 1a). Alignments between *NAT8/NAT8B *and their flanking areas (web-based global alignment tool CLUSTALW [17]) revealed a high nucleotide homology between extended genomic regions. The forward primer for *NAT8 *was positioned within a 313 bp *NAT8*-specific insertion (Figure 1b). The Primer3 primer designing software [18] was used to search for the matching reverse primer from the region upstream of *NAT8*, where the sequence homology with *NAT8B *region did not continue (> 2722 bp upstream; Figure 1b). The uniqueness of long-range PCR primers was checked using BLAST [19]. PCR reactions were performed using Long PCR Enzyme Mix (MBI Fermentas, Vilnius, Lithuania) and Smart-Taq Hot DNA polymerase (Naxo, Estonia) according to the standard protocol recommended by the manufacturers. Detailed conditions for long-range (10341 bp) and nested (2322 bp) PCR, product purification, sequencing, sequence contig assembly and polymorphism identification are described in detail elsewhere [20]. The resequenced region covered 2185 bp including the entire *NAT8 *(1569 bp) as well as 541 bp of the 5' upstream and 75 bp of the 3' downstream regions. All primers used for the *NAT8 *PCR amplification and resequencing are listed in Table 1.

**Table 1:** PCR and resequencing primers for human *NAT8*

**Primer ID**	**Primer sequence 5'-3'**
***NAT8 *resequencing**	
	
**Long-range PCR primers**	
NAT8_10kb_F	TGTAATCCCACACTTTTAGAGGCCAAG
NAT8_10kb_R	GAGTAGGTAAACAAAGCAGCCAGGAA
	
**Nested PCR primers**	
NAT8_2kb_F	GCAAAATTGCTGACTTCAGACTC
NAT8_2kb_R	CAACAGCCAGAACACACACAAG
	
**Sequencing primers**	
NAT8_SEQ_1F	TGCAACATTATGCTGGCACT
NAT8_SEQ_2F	CAGACTCAGCCACCCAGAAG
NAT8_SEQ_3F	AGGGTCAGAAAGACCTGCATT
NAT8_SEQ_4F	GCATCCAGGAGATACCGTCT
NAT8_SEQ_1R	ATTGCTGAAGCTGCCTCGAAC
NAT8_SEQ_2R	AGGTAGGTGCCCCCTCAAT
NAT8_SEQ_3R	CCTGAACAGGCAAGAGAGGT
NAT8_SEQ_4R	GTCAAGCCGAATGTCAACCT

In the extended association study of the 5'upstream SNPs, only the sequencing primers 4F and 4R were used. For the comparison of sequence diversity parameters, we resequenced (n = 14) the promoter regions (+100 bp up to -500 bp relative to mRNA transcription initiation site) for 29 previously established blood pressure associated genes from literature: *ACE, ADD1, ADD2, AGT, AGTR1, AGTR2, KCNJ1, CYP17A1, NPR1, CLCNKA, HSD11B1, CYP11B2, CLCNKB, KLK1, NPR2, BSND, NR3C2, CYP21A2, SAH, SGK, SLC12A3, SCNN1A, IL1A, SLC14A2, SLC22A2, REN, ATP1A, SCNN1B *and *SLC8A1 *(primers used for PCR and resequencing, see additional file 1: Primers).

### Subjects for Association Studies

#### Association study for blood pressure

Long-term blood donors (n = 137; 76 males, 61 females; age 40 ± 7.8 years; BMI 25 ± 2.6) were recruited in the framework of HYPEST study (participation rate 51.5%; Org et al., unpublished data) from the North Estonian Blood Center [21] coordinating the blood donation in northern, eastern, and western Estonia, and the Blood Centre of the Tartu University Clinics [22] coordinating the blood donation activity in central and southern Estonia. The exclusion criteria for donors included cardiovascular diseases, diabetes and antihypertensive treatment ascertained by confidential self-reported signed questionnaires and medical examination. Both Blood Centres have standardized protocols for measuring blood pressure by responsible trained clinicians. After a rest in a sitting position, the blood pressure reading of a donor was obtained during each visit to the Blood Donation Centre. All recruited individuals possess a documented history of multiple systolic and diastolic blood pressure (SBP, DBP) readings (on average 4 readings per individual, range 2–14) during 3 ± 2.5 years (range 1–7 years). Most of the BP measurements per subject have been obtained using a standard mercury column sphygmomanometer and size-adjusted cuffs. Occasionally, outside of blood donation centres BP of the participants was measured by oscillometric method using NAIS DIAGNOSTEC blood pressure watch (Matsushita Electronic Works) after a rest in a sitting position and with the arm placed at the heart level. No systematic deviation was observed between the two devices. The BP parameters across the whole cohort study group were as following: SBP 134.2 ± 11.5 mmHg within a range of 113.5 – 171 mmHg; and DBP 84.6 ± 7.6 mmHg within a range of 65.5 – 110 mmHg. Quantitative analysis was performed with a calculated median of a subject's BP readings adjusted to age and sex as co-variables.

#### Association study for estimated glomerular filtration rate (eGFR)

The study group of long-term essential hypertension (EH) patients (n = 157; 78 males, 79 females; age at the time of EH diagnosis 47 ± 9.7 years; age at recruitment 59 ± 9 years; BMI 28 ± 3.8) without and with hypertensive nephropathy were recruited (participation rate 42.3%) by blood pressure specialists during the patients' ambulatory visit or hospitalization at the North Estonia Medical Center, Centre of Cardiology (cardiologist M.V.) or at the Cardiology (coordinated by G.V.) and Internal Medicine Clinics (nephrologist M.R.), Tartu University Hospital, Estonia. The selection criteria for the study group were as following:

- Long term (mean duration 12.5 y) essential hypertension diagnosed by a clinical specialist (SBP > 140 mmHg, DBP > 90 mmHg).

- Exclusion of patients with diabetes and primary renal failure by a clinical specialist

- All recruited hypertension patients received antihypertensive treatment

Diagnose of hypertensive nephropathy was assigned by nephrologists (M.R.) based on longstanding blood pressure elevation together with proteinuria and presence of progressive chronic kidney disease. Differential diagnosis was established using methods *per exclusionem *i.e. the patients possessed no symptoms of glomerulonephritis, systemic diseases, amyloidosis or other glomerulopathies, confirmed by diagnostic labororatory tests.

The venous blood for serum biomarker analysis was drawn in the morning after an overnight fast. Urea (measurement uncertainty 9.7%), creatinine (m.u. 17.2%) and albumine (m.u. 6.9%) in the serum were measured by standardized assays (Cobas Integra 800^® ^analytical platform, Roche Diagnostics, Inc.) at the United Laboratories, Tartu University Clinics [22] or at the Diagnostics Division Laboratory, the North Estonia Medical Centre [23]. The tests of for the serum biomarkers have been accredited by the Estonian Accreditation Centre [24] according to the standards of the European co-operation for Accreditation [25]. Measurement uncertainty was estimated using EURACHEM guidelines [26]. It is defined as the parameter associated with the result of a measurement that characterizes the dispersion of the values that could reasonably be attributed to the measurand (e.g. the concentration of a biomarker).

Kidney function was evaluated using estimated glomerular filtration rate (eGFR), the most recommended overall index of kidney function in health and disease [27]. The formula [28] includes both, demographic (age, sex, race) and blood serum (urea – SUN [mg/dl], creatinine – P_Cr _[mg/dl], albumin – Alb [g/dl]) variables.

eGRF (ml/min/1,73 m^2^) = 170× (P_Cr_)^-0,999 ^× (Age)^-0,176 ^× (0,762 if patient is female) × (SUN)^-0,17 ^× (Alb)^+0,318^

A decrease of eGFR below 60 ml/min/1.73 m^2 ^precedes the onset of kidney failure (K/DOQI 2002). The distribution of eGFR values for our study population was 72 ± 28.5 (range 5 – 143).

### Statistical analysis

Methods used for Hardy-Weinberg equilibrium estimations, sequence diversity calculations and *in silico *prediction of transcription factor binding sites are described in detail elsewhere [29,30]. Quantitative genetic association analyses for blood pressure and serum biomarkers were performed with association analysis toolset PLINK [31]. Association was tested using linear regression analysis with additive effect model and likelihood-ratio test (LRT) with age, sex and BMI as co-variables. Statistical significance for the different distribution of the quantitative measurements (BP, GFR) in two subgroups based on the genotype was verified using non-parametric Mann-Whitney two-sided U-test performed with a web-based interface [32]. In all computed statistical tests a p-value < 0.05 was considered statistically significant and p < 0.1 as suggestive.

## Results

### Detailed diversity patterns of NAT8 by resequencing

**Figure 2:**
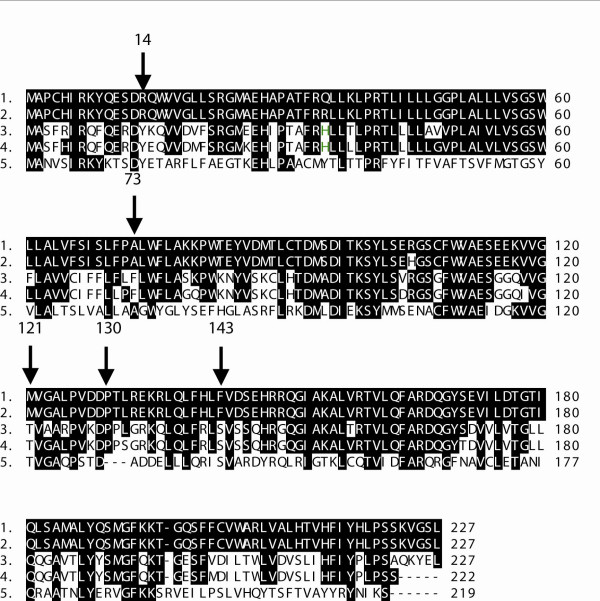
**The alignment of NAT8 protein sequences: 1- *Homo sapiens*, 2- *Pan troglodytes*, 3- *Mus musculus*, 4- *Rattus norvegicus*, 5-*Xenopus tropicalis*.** Arrows pointing at polymorphic amino acid positions (14 Arg-His, 73 Ala-Val, 121 Met-Thr, 130 Pro-Pro, 143 Phe-Ser).

To uncover the detailed genetic variation of a putative human blood pressure candidate gene *NAT8 *(*N-acetyltransferase 8*; 2p13.1-p12) we resequenced the gene in a random Estonian population sample (n = 42). The analyzed region (1569 bp of the entire *NAT8*; 541 bp of 5' upstream and 75 bp of 3' downstream) harboured 15 SNPs including 10 rare (Minor allele frequency, MAF < 10%) and five common (MAF > 10%) polymorphisms (Table 2). Nine SNPs (n = 6 in 5'upstream region; n = 1 in intron 1, exon 2 and 3'downstream region) were previously characterized in dbSNP database, including five HAPMAP SNPs (rs482951, rs2280506, rs2280507, rs6744273, rs13538). Six rare variants are novel, neither represented in dbSNP nor reported elsewhere: one SNP in untranslated exon 1, one in intron 1 and four nonsynonymous substitutions in exon 2. The alignment of NAT8 protein sequences (Figure 2) of several vertebrate species (*Pan troglodytes, Mus musculus, Rattus norvegicus, Xenopus tropicalis*) indicated that the human polymorphic amino acid positions are not conserved in evolution. Thus we did not include these SNPs for further association study.

**Table 2:** *NAT8 *genomic region SNPs identified by resequencing in random Estonian population samples (n = 42)

**Region**	**Location^a^**	**dbSNP rs#**	**SNP^b^**	**Codon change**	**MAF**
5' upstream	-1255	rs4852954	T/C	-	0.25
	-1153	rs4852953	A/G	-	0.226
	-1152	rs4852952	C/T	-	0.226
	-1041	rs4852951	G/C	-	0.238
	-911	rs2280506	T/C	-	0.083
	-803	rs2280507	C/T	-	0.071
Exon1	-763	-	T/C	-	0.012 (S)
Intron1	-365	rs6744273	A/G	-	0.071
	-222	-	T/G	-	0.012 (S)
Exon2	41	-	C/T	Arg-His	0.024
	218	-	G/A	Ala-Val	0.012 (S)
	362	-	A/G	Met-Thr	0.012 (S)
	390	-	G/T	Pro-Pro	0.012 (S)
	428	rs13538	A/G	Phe-Ser	0.167
3'downstream	859	rs1879662	G/A	-	0.071

Abbreviations: *MAF*, minor allele frequency; *S*, singletons

^a^Location relative to ATG

^b^Major/minor allele

The *NAT8 *resequencing data deviated strongly from the polymorphism pattern in public dbSNP database [33] with 39 SNP entries for *NAT8 *region (Figure 1c). The higher number of SNPs in dbSNP might represent paralogous sequence variants (PSVs) between *NAT8 *and its duplicate gene *NAT8B *as all the unidentified dbSNP entries co-located with the sequence positions where *NAT8 *and *NAT8B *diverge (see additional file 2: Alignment).

Direct estimation of per-site heterozygosity (π) was derived from the average pairwise sequence difference. The *NAT8 *gene (1569 bp; π/bp = 0.00037) and mRNA (953 bp; (π/bp = 0.00045) exhibit two times lower sequence diversity compared to a data set of 74 genes [34] (π/bp = 0.00080 for European Americans; Table 3). Tajima's D (D^T^) statistic was used to estimate the difference between the parameters π and θ, representing the expected per-site heterozygosity based on the number of segregating sites. In case of neutrality, π equals θ and thus D^T ^statistic equals zero. For *NAT8 *coding region, the calculated negative D^T ^values refer to the excess of rare SNPs suggesting a purifying selection pressure. In contrast, the *NAT8 *5'upstream region exhibits approximately eight times higher diversity (π/bp = 0.00320; Table 3) and a positive D^T ^value indicating an excess of common SNPs. Accordingly, four of the five *NAT8 *SNPs with minor allele frequency > 10% are located in the upstream region. Notably, this high diversity is uncommon in the dataset of promoter regions (+100 bp to -500 bp relative to mRNA transcription start) of 29 previously studied blood pressure genes resequenced to compare with *NAT8 *(Table 3). The diversity level of *NAT8 *upstream area (6 SNPs) exceeded about eight times the average of 29 reference genes (π/bp = 0.00320 versus π/bp = 0.0004). Of the 29 studied gene upstream regions, 17 (59%) had no SNPs, in the rest of the genes (n = 12) the number of SNPs ranged from one to four with the heterozygosity parameter (π) being 0.00023-0.0017. Interestingly, similarly to *NAT8*, several genes with the most variable upstream regions (*CYP11B2, IL1A, SLC14A2*) have also duplicate copies.

**Table 3:** Diversity parameters for resequenced *NAT8 *gene and promoter regions of 29 genes associated with blood pressure determination

**Region**	**SNPs**	**π**	**θ**	**D^T^**
A. *NAT8 *region				
Gene (1569 bp)	9	0.00037	0.00102	-1.58
mRNA (953 bp)	6	0.00045	0.00127	-1.49
Promoter (600 bp)^a^	6	0.00320	0.00221	1.05
B. Promoter regions (600 bp)^a ^of 29 blood pressure associated genes^b^				
*AGTR1*	1	0.00082	0.00043	1.47
*CYP17A1*	2	0.00058	0.00043	0.57
*CLCNKA*	2	0.00081	0.00086	-0.11
*CYP11B2*	3	0.00168	0.00085	2.02
*NPR2*	1	0.00042	0.00043	-0.018
*BSND*	2	0.00023	0.00043	-0.74
*SAH*	2	0.00071	0.00087	-0.38
*SGK*	1	0.00079	0.00043	1.36
*SLC12A3*	1	0.00075	0.00043	1.216
*SCNN1A*	3	0.00167	0.00128	0.721
*IL1A*	2	0.0017	0.00086	2.02
*SLC14A2*	4	0.00133	0.00085	1.17

Abbreviations: *π*, estimate of nucleotide diversity per site from average pairwise difference among individuals; *θ*, estimate of nucleotide diversity per site from number of segregating sites; *D*^*T*^, value of Tajima's D statistics expressing the difference between π and θ estimates

^a^Region between -500 bp and +100 bp relative to mRNA transcription start point

^b^No SNPs were detected in Estonian sample by resequencing the promoter regions for *ACE, ADD1, ADD2, AGT, AGTR2, KCNJ1, NPR1, HSD11B1, CLCNKB, KLK1, NR3C2, CYP21A2, SLC22A2, REN, ATP1A1, SCNN1B, SLC8A1 *genes

### Association study of NAT8 5' upstream SNPs with blood pressure and eGFR

**Figure 3:**
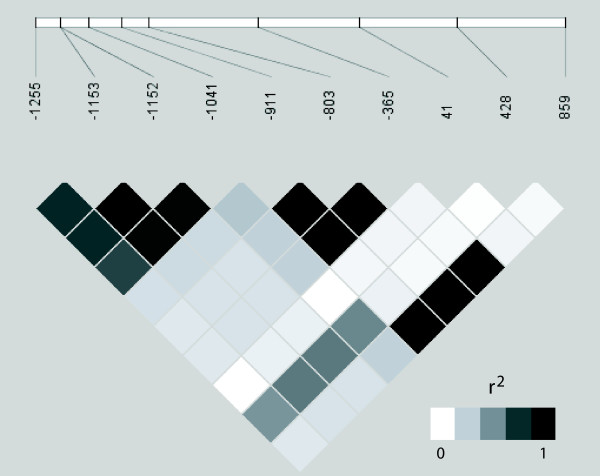
**LD structure of the resequenced *NAT8 *genomic region shown as r^2^-blot.** Upper white bar depicts the positions of identified SNPs (Table 2) relative to the location of *NAT8 *gene ATG (A denoted as +1). Singleton polymorphisms were excluded from calculations.

**Figure 4:**
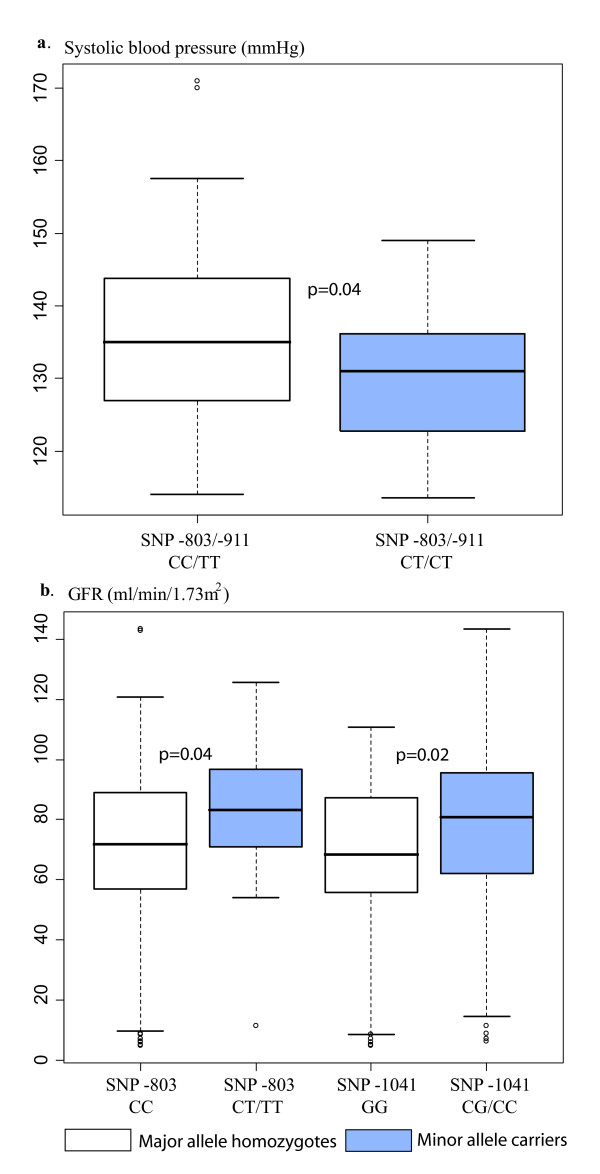
**Notched box plots for the distribution of (a) systolic blood pressure (SBP) readings; and (b) estimated glomerular filtration rate (GFR) values for the major allele homozygotes and the minor allele carriers of *NAT8 *promoter SNPs.** The boxes represent the 25^th ^and 75^th ^percentiles. The median is denoted as the line that bisects the boxes. The whiskers are lines extending from each end of the box covering the extent of the data on 1.5 × interquartile range. Circles represent the outlier values. P-values from Mann-Whitney U-test are shown, applied to test the statistical difference in the distributions of SBP or GFR estimates between two groups.

A *NAT8 *5'upstream putative promoter region [SNPs -1255 (T/C), rs4852954; -1153 (A/G), rs4852953; -1152 (C/T), rs4852952; -1041 (G/C), rs4852951; -911 (T/C), rs2280506 and -803 (C/T), rs2280507] was targeted in association studies for BP and estimated glomerular filtration rate (eGFR). Despite close vicinity, these SNPs form two distinct allelic association blocks: from -1225 to -1041 and from -911 to -365 (Figure 3). Notably, the latter region is also in LD with the 3' downstream SNP (+859 G/A; rs1879662). We used median values of systolic and diastolic blood pressure (SBP, DBP) readings in a cohort of long-term healthy Estonian donors (n = 137) with no history of cardiovascular diseases and respective treatments. SBP was significantly associated with *NAT8 *SNPs -803 (C/T) and -911 (T/C) using LRT (p = 0.028) and linear regression model (additive effects of allele dosage; p = 0.015) tests (Table 4). The significant negative regression coefficient (-5.034) referred to the association of the minor alleles of SNP -803/-911 with lower SBP values and indicated that these variants may possess a potential protective effect towards hypertension. Indeed SBP was significantly (p = 0.04; Mann-Whitney U test) lower by average 5 mmHg for the carriers of the minor alleles (median 131 mmHg; mean 130.5 ± 10.0; n = 33) compared to the major allele homozygotes (median 135 mmHg; mean 135.5 ± 11.7; n = 104) for SNPs -803/-911 (Figure 4a), supporting a protective effect of these alleles against blood pressure elevation. Association analysis of SBP with other upstream SNPs and of DBP with any *NAT8 *SNPs did not reveal any statistical significance (p = 0.1).

**Table 4:** P-values from association tests of *NAT8 *upstream SNPs: linear regression (additive model) adjusted for age, gender and BMI

	**Blood pressure**		
		
**SNP ID**	**SBP**	**DBP**	**eGFR**
-803 (C/T)	**0.015**	0.157	**0.042**
-911 (T/C)	**0.015**	*0.100*	*0.060*
-1041 (G/C)	0.519	0.943	**0.050**
-1152 (C/T)	0.373	0.828	*0.081*
-1153 (A/G)	0.373	0.828	*0.081*
-1255 (T/C)	0.659	0.870	0.070

Abbreviations: *SBP*, systolic blood pressure; *DBP*, diastolic blood pressure; e *GFR*, estimated glomerular filtration rate; **p < 0.05; ***p ≤ 0.1*.

We further tested the association of *NAT8 *upstream SNPs with the reduction of kidney function as a consequence of essential hypertension using an Estonian cohort of long-term hypertension patients without and with hypertensive nephropathy. Linear regression analysis (additive model) and LRT test detected significant (SNP-803 and SNP-1041; p < 0.05) and borderline (p ≤ 0.08) associations of *NAT8 *upstream SNPs with eGFR estimates reflecting the efficiency of kidney function (Table 4). Significant positive linear regression coefficients indicated the association of SNP-803 (10.7) and SNP-1041 (6.4) with higher GFR values. The distribution of eGFR values for heterozygotes of SNP -803 (median 83.3; mean 82 ± 21.5; n = 33) and SNP -1041 (median 80.5; mean 77 ± 28; n = 90) was significantly higher (Mann-Whitney U test, p = 0.04 and p = 0.02) than for wild-type homozygotes (SNP -803: median 71.9; mean 70 ± 29.7, n = 124; SNP -1041: median 68.3; mean 66 ± 28; n = 67) on average 12 and 11 GFR units, respectively (Figure 4b).

### Functional significance of NAT8 5'upstream SNP alleles

Possible functional significance of *NAT8 *upstream SNP alternative alleles was targeted with the *in silico *transcription factor binding site (TFBS) prediction using MatInspector [35] software. Several potential TFBSs within predicted classical promoter region (+100 to -500 bp relative to mRNA transcription start) are missing on the chromosomes that carry the minor alleles for *NAT8 *upstream SNPs -911 (major allele T/minor allele C), -1152 (C/T) and -1153 (A/G) (Figure 1d). Compared to the common T variant, the C allele of SNP -911 lacks potential binding sites for seven competing or co-activating TFs, including factors involved in inflammatory, cardiovascular or renal function: oestrogen related receptor (ERR) [36], RAR-related orphan receptor alpha1 (RORA) [37], HMG box-containing protein 1 (HBP1) [38], nuclear factor kB (NF-kB)/c-rel [39]. Allele- specific binding of ERR was confirmed by another TFBS prediction program AliBaba 2.1. [40]. Minor combinatory variant (T-G) for neighbouring SNPs -1152/-1153 miss two potential TFBSs, one for serum response factor (SRF) and the other for myeloid zinc finger protein (MZF1) located on the opposite strands. SRF is a transcription factor conserved from flies to humans and shown to regulate coordinated gene expression in cardiac and vascular smooth muscle cell differentiation [41].

## Discussion

We report the first study targeting human *N-acetyltransferase 8 *(*NAT8*) as a potential blood pressure (BP) and kidney function associated gene. Although the knowledge about *NAT8 *is scarce, it was shown to be highly expressed in developing as well as adult human kidney and liver [14]. A recent study associated *NAT8 *expressional profile with nephrotoxicity response [15]. Kidney function has been associated with BP from two complimentary viewpoints: (1) link between elevated blood pressure and risk for kidney failure [4,6]; and (2) link between renal structural/functinonal abnormalities developed during intrauterine life as susceptibility to hypertension in adult life [7,10]. In listing the candidate genes for association studies of human blood pressure traits, the genes protecting and contributing to normal fetal renal programming have often been overlooked as potential susceptibility factors.

We showed that the putative upstream *NAT8 *promoter region is highly diverse compared to the gene's coding region, as well as to the promoters of BP-associated reference genes resequenced for comparison. High diversity may indicate the lack of selective constraint and/or the maintenance of several functioning promoter variants with differential effect on gene expression. For a number of genes shown to contribute to blood pressure regulation, the promoter polymorphisms have been associated with the diagnosis of hypertension. Examples of experimentally confirmed differential effects of alternative alleles on promoter activity are *CYP11B2 *[42-44], *NPR1 *(reviewed in [45]), *SCNN1A *[46] and *AGT *[47].

In a pilot association study based on a random Estonian population cohort we identified an association between *NAT8 *promoter SNPs and systolic BP. Consistently, in a separate cohort of essential hypertension patients the same markers showed association with estimated glomerular filtration rate (eGFR). Interestingly, the minor alleles of the same SNPs reveal protective effects towards both, the elevated BP and the risk of kidney failure as the complication to high blood pressure. We are aware that the results of these pilot association studies may be biased due to phenotypic misclassification and/or may be underpowered due to limited sample sizes. Also, multiple testing tends to inflate type 1 error rates. Another limitation may be the lack of sensitivity of the eGFR calculations based on serum creatinine instead of the actual GFR values obtained from measurements using an exogenous filtration marker. Thus, replication of the association of *NAT8 *with SBP and eGFR in other study populations, as well as functional experiments addressing the effect of the alternative promoter variants are required to ultimately determine the role of this gene in blood pressure regulation and kidney function. While evaluating the data from the association studies, one also has to keep in mind that both BP regulation and kidney metabolic function are complex traits determined by the combination of environmental, individual's life style and heterogenous genetic factors and thus the contribution of each gene variant is expected to be small. Recently, Yamada et al [48] performed a large association study with 150 SNPs in 128 functionally relevant BP regulating genes for 2818 hypertensive Japanese and 2035 matched controls. Using multivariable logistic regression analysis, significant associations (0.002 < p < 0.05) with hypertension were detected only with 10% (n = 15) of the targeted SNPs. Interestingly, seven of these were promoter variants and in 10 SNPs out of the significant 15 the carrier status of the minor allele provided a protective effect against blood pressure elevation.

High homology between *NAT8 *and its pseudogene *NAT8B *is due to a recent origin of *NAT8B *through the duplication of an ancestral gene after the ape-Old World monkey split. In chimpanzee both gene copies are still active, whereas in human *NAT8B *appears to be inactive due to human-specific nonsense mutations [49]. There are multiple examples of gene conversion events between genes and their pseudogenes causing the dysfunction of the genes [50]. In case of *NAT8*, the alternative promoter sequence may have arisen through gene conversion from *NAT8B *leading to differentially expressed gene variants.

Although the exact function of *NAT8 *is still to be determined, it has recently been shown to participate in detoxification processes of TCE (trichloroethylene) metabolite DCVC (S-(1,2-dichlorovinyl)-L-cysteine) in the kidney. DCVC detoxification in kidneys has two alternative metabolic pathways: (1) bioactivation through B-lyase activity leading to the accumulation of a nephrotoxic reactive sulfhydryl-intermediate (DCVSH – dichlorovinyl mercaptan) and (2) N-acetylation of DCVC resulting in a harmless acetylated product (NacDCVC – N-acetyl-S-(1,2-dichlorovinyl)-L-cysteine) excreted in urine [51]. When human renal proximal tubule cells were exposed to low concentrations of DCVC, *NAT8 *expression was reduced more than twice [15]. We hypothesize that the transcriptional activation of the common *NAT8 *promoter variant is repressible by toxic substances (e.g. TCE metabolites) and reduction of N-acetylation activity may lead to favouring of the nephrotoxic pathway. Due to differential cocktail of activating transcription factors for the *NAT8 *promoter variant with alternative SNP alleles, the minor *NAT8 *variant may be less sensitive to transcriptional suppression and the carriers may be better protected for the nephrotoxicity. The two disease processes, renal response to nephrotoxins and the pathogenesis of hypertension may be connected through overlapping physiological pathways. A representative example is the enzyme haem oxigenase 1 (HO-1) responsible for the catabolism of free heam, being induced in some models of hypertension as well as up-regulated in a variety of kidney injury models, including the effect of nephrotoxins (reviewed in [52]).

## Conclusion

The full resequencing and pilot association study of a novel positional candidate gene for blood pressure and renal function, human *N-acetyltransferase 8*, suggested the contribution of highly variable *NAT8 *promoter polymorphisms in determination of systolic blood pressure and estimated glomerular filtration rate. Based on *in silico *analysis, we raise the hypothesis that the alternative SNP alleles within *NAT8 *upstream region may have differential effect on gene expression.

## Competing interests

The author(s) declare that they have no competing interests.

## Authors' contributions

PJ designed the study, performed resequencing, and contributed to the analysis and interpretation of the data as well as to writing of the manuscript. EO, MV, GV and MR recruited hypertensive individuals for the study, collected the epidemiological data, and revised the manuscript since its early versions. EO, KK, GV and PK assisted in collection of long-term blood donors, preparation of the DNA samples and laboratory experiments. ML contributed in outlining the study design and directing research, participated in the data analysis and drafted the manuscript. All authors read and approved the final manuscript.

## Pre-publication history

The pre-publication history for this paper can be accessed here:



## Supplementary Material

Additional file 1 PCR and resequencing primers for the promoter regions (+100 bp up to -500 bp relative to mRNA transcription initiation site) of 29 genes associated with blood pressure determination. Click here for file

Additional file 2 Nucleotide sequence alignment between human *NAT8 *gene and its duplicated pseudogene *NAT8B*. The alignment includes the entire gene along with 541 bp of 5' upstream and 700 bp of 3' downstream regions. Untranslated mRNA and protein-coding regions are marked with thin and bold lines, respectively. Nucleotides typed in bold indicate SNPs detected by resequencing and their position relative to ATG. Divergent positions in *NAT8 *and *NAT8B*, which co-localize with human *NAT8 *SNPs in NCBI dbSNP, are boxed. Click here for file
