# Supplementary material for: N-acetyltransferase 8, a positional candidate for blood pressure and renal regulation: resequencing, association and in silico study
Source: BMC Med Genet. 2008 Apr 10;9:25. doi: 10.1186/1471-2350-9-25 (PMC2330028; doi:10.1186/1471-2350-9-25)
Supplement: Additional file 1 — PCR and resequencing primers for the promoter regions (+100 bp up to -500 bp relative to mRNA transcription initiation site) of 29 genes associated with blood pressure determination. [file 1471-2350-9-25-S1.pdf]

Additional file 1

File format: pdf

Title and description of the data:

PCR and resequencing primers for the promoter regions (+100 bp up to –500 bp relative to mRNA transcription initiation site) of 29 genes associated with blood pressure determination. F – forward primer; R – reverse primer

Primer ID	Primer sequence 5´-3´
**PCR primers**	
ACE_PCR_F	TGATGTTTTCTGCTGAGCGACTGG
ACE_PCR_R	ATTCTCCGCGGTGATGTTGGTGT
ADD1_PCR_F	ATTTGTCTCGCTGCTTGTTT
ADD1_PCR_R	CTAGCTAGCCTCACCTCAGA
ADD2_PCR_F	ATCTTAGCTATCTCCGGTCGGCCA
ADD2_PCR_R	ATTCTGGTTCCCTATCCAGTTCCCCA
AGT_PCR_F	TTGGCCATGATGACTCTGTTCCTG
AGT_PCR_R	GCCTGCATCCTGAAGGCATTTTGT
AGTR1_PCR_F	TCTGTAGTCTCGTGATGTCTTTATCTG
AGTR1_PCR_R	GGACACCGTGACATGAAACTTAGC
AGTR2_PCR_F	TCACTCTTGCTTCTGCTGGATTGG
AGTR2_PCR_R	GCTTGCTTAGTGCCTAAACACACTCC
ATP1A1_PCR_F	AAGTCACAGGAGGCACTCTGA
ATP1A1_PCR_R	GTCCTGTCGCTGGAGAATCA
BSND_PCR_F	TGGAGACAGCACCAAATCTGGGA
BSND_PCR_R	ACAATGAAGCCGATCCGGAAGGT
CLCNKA_PCR_F	TTAATCTCCATGGTAAACCCCGGC
CLCNKA_PCR_F	TGGACAAATGACAGGAGGGTGGTT
CLCNKB_PCR_F	TTCCTGACCCCAATTCACTGCCTT
CLCNKB_PCR_R	ACTGCAGAGTCCCATGGATGGCT
CYP11B2_PCR_F	TTTACCTGAAAATGGGCCCCAGC
CYP11B2_PCR_R	TGGAAAAGGCCAAAGCATGGTG

CYP17A1_PCR_F	TGGTGGCCGACAATCACTGTAGTCTT
CYP17A1_PCR_R	AATGTTATGAAACGGCCTCCCACC
CYP21A2_PCR_F	TCAACCAAAGAAAGGTGCTCCTGG
CYP21A2_PCR_R	CGAATTTCTGAGTCAGGCCAAGCA
HSD11B1_PCR_F	CAGCTGATGCAGTCACTAAAATG
HSD11B1_PCR_R	CAAGAGACTTCTGGTTCCATCTG
IL1A_PCR_F	CTTCTGTAGGATATGCCCAAGGT
IL1A_PCR_R	GAGGAACCAAGGTAAGCAGAAAT
KCNJ1_PCR_F	GATCAAAAGCATCCAGTGAGTGGCTC
KCNJ1_PCR_R	TCTTGCATCAAAGGTGCAGGGACT
KLK1_PCR_F	TGGGAGTGAGCACTATGACAACATGG
KLK1_PCR_R	TTCACATTCCTGGATCTGGAGGCA
NPR1_PCR_F	TTCAGAGGGTCCTGTCCTCCAAA
NPR1_PCR_R	GTTGTGCTCCCACTTGAGGTCCA
NPR2_PCR_F	AGAAGCGGAGCAGAGAGGAG
NPR2_PCR_R	GGTCATGGTACAGCTTGAGGTC
NR3C2_PCR_F	TCCGGATTACGCAGCCACTTCTC
NR3C2_PCR_R	AACCTCGAGGGTCGGGTGAA
REN_PCR_F	AGCTGATCGTAAGGACTGAATGA
REN_PCR_R	ATGCTAAGTCTCCCTCCACTCTC
SAH_PCR_F	TGAGGAAAGAGTTCCAGTCAGTG
SAH_PCR_R	TTTGTGTGTGCATACTCCTCATC
SCNN1A_PCR_F	CTGCCTCCTCTCTCTAATCCTG
SCNN1A_PCR_R	ACTTAGTGAGCGGGGAGGAGAC
SCNN1B_PCR_F	GAGGCTAGAGCACAGGTGTAGTG

SCNN1B_PCR_F	CTCAGACAGGCACCCAAGC
SGK_PCR_F	CTGTGGATGAAGGAGGAGAAATA
SGK_PCR_R	AGCGAGTCCTTCCTGCTGAG
SLC12A3_PCR_F	AGATCGAGCCCTGACCTAATCG
SLC12A3_PCR_R	GACAGCACTTACCTTGAGGAAGGA
SLC14A2_PCR_F	TTGTCTAACCAGAAGCCAAATG
SLC14A2_PCR_R	CAAAATTGTGCCTTAGCAACAG
SLC22A2_PCR_F	AAAAAGTGAAACTCCCCTCCAT
SLC22A2_PCR_F	GACACAAAGATAGTGGCTTGGAC
SLC8A1_PCR_F	GCAAAATCACCCCTTTCTTACAG
SLC8A1_PCR_F	CCAAGTAAGTGTGGAGCAATAGG
**Sequencing primers**	
ACE_SEQ_F	GTTTTCTGCTGAGCGACTGG
ACE_SEQ_R	GTTCGGCGCTGGAGTTGTAG
ADD1_SEQ_F	CATTTGTCTCGCTGCTTGTTT
ADD1_SEQ_R	CTAGCTAGCCTCACCTCAGAAGC
ADD2_SEQ_F	CTAATCTGCAGGGCAGCGTTTT
ADD2_SEQ_R	GAGGAGAATGTAGTGAGAGTTGGA
AGT_SEQ_F	CCTCCTGTAAGACCCCAGGT
AGT_SEQ_R	GTGTTTAACAGTCTCCCCAGCTA
AGTR1_SEQ_F	GTCTCGTGATGTCTTTATCTGGTTT
AGTR1_SEQ_R	CTTGAAGTCCCCCAAGCAT
AGTR2_SEQ_F	AACCACAAATATGACAGAGACC
AGTR2_SEQ_R	CCTGTAAGAGAAACAGCAGCTAAA
ATP1A1_SEQ_F	TAAAGAGGACCCCAAGTCACAGG

ATP1A1_SEQ_R	GAGCCAAGTGGAGGGAGCTA
BSND_SEQ_F	CACCAGAGCCACGCTTTT
BSND_SEQ_R	GTAGTTCAGCCTAAACACCGAGA
CLCNKA_SEQ_F	CTGTGAGAGGAGGGCCAGTT
CLCNKA_SEQ_F	ACCTGGCTCTCACCTTGGAT
CLCNKB_SEQ_F	CACCTCCCTGAGACACCATT
CLCNKB_SEQ_R	TCTGACTCTCCCTGTCCACCTA
CYP11B2_SEQ_F	CCAGGTGCAGGTGCTCATA
CYP11B2_SEQ_R	CTGCAAGGAGGGATACAAATTAC
CYP17A1_SEQ_F	GCCCATACGAACCGAATAGAT
CYP17A1_SEQ_R	CCCTTTCGTTCCCATACACA
CYP21A2_SEQ_F	CTCCACAACATGCGAACAATAC
CYP21A2_SEQ_R	GAGCTTCCACCAGTTCCACA
HSD11B1_SEQ_F	GCTGATGCAGTCACTAAAATGGT
HSD11B1_SEQ_R	AGCCCTCCTGTTCATTTCGATAG
IL1A_SEQ_F	CTTCTGTAGGATATGCCCAAGGT
IL1A_SEQ_R	AGCTTCTACCCTAGTCTGGTGCT
KCNJ1_SEQ_F	GGTCCATAGCACAATCACTATGT
KCNJ1_SEQ_R	GGAGAAACACCTCATTGTCTCTTT
KLK1_SEQ_F	ATGAGGGTGGGGTGTTGGA
KLK1_SEQ_R	CTGGAGGCACTAGGAATGTG
NPR1_SEQ_F	GTCCTGTCCTCCAAAGAGGTAG
NPR1_SEQ_R	GACCAGGGGTACGAGGTATTG
NPR2_SEQ_F	CTCCGGGAGAATCCTTCAC
NPR2_SEQ_R	AGCGGTGCCAGGTACTCAGA

NR3C2_SEQ_F	GTCTCTCGCCGTCTACCTGTT
NR3C2_SEQ_R	GAGGTAACTCTGGAGACAGTGGA
REN_SEQ_F	AGCTGATCGTAAGGACTGAATGA
REN_SEQ_R	GGGTTTCACCATTGTTGGTC
SAH_SEQ_F	CTGTGCAGAGTGAGGCAGA
SAH_SEQ_R	GCCCAAGACTTTAATGACAGC
SCNN1A_SEQ_F	ACCTCGAGCTGTGTCCTGAT
SCNN1A_SEQ_R	GAGGGTGAGGCTGACCTGT
SCNN1B_SEQ_F	GGTGGGCTGTTTGAATTTCT
SCNN1B_SEQ_F	GAAACTGAGGCCGGGACAG
SGK_SEQ_F	AATTTCCACTTTGCGTCTCCT
SGK_SEQ_R	GCTTTCGTCGGAGTTCTCG
SLC12A3_SEQ_F	ATCCCTGTCCTGTTTTGTTCCAA
SLC12A3_SEQ_R	ACATCGATCGTGTTGTAGCCAAA
SLC14A2_SEQ_F	CCTGCAGGTGCACAGAAGT
SLC14A2_SEQ_R	CCAATGTGGTGAAACCCTGT
SLC22A2_SEQ_F	GCTCCAGGACATCGTCCAC
SLC22A2_SEQ_F	GCCTGCATTTACACTTGACCT
SLC8A1_SEQ_F	TTTCCTTCTCCTTCCATTTCTG
SLC8A1_SEQ_F	TAAGATGCTCTCTGGCGTTTAAG
