# Supplementary material for: N-acetyltransferase 8, a positional candidate for blood pressure and renal regulation: resequencing, association and in silico study
Source: BMC Med Genet. 2008 Apr 10;9:25. doi: 10.1186/1471-2350-9-25 (PMC2330028; doi:10.1186/1471-2350-9-25)
Supplement: Additional file 2 — Nucleotide sequence alignment between human NAT8 gene and its duplicated pseudogene NAT8B. The alignment includes the entire gene along with 541 bp of 5' upstream and 700 bp of 3' downstream regions. Untranslated mRNA and protein-coding regions are marked with thin and bold lines, respectively. Nucleotides typed in bold indicate SNPs detected by resequencing and their position relative to ATG. Divergent positions in NAT8 and NAT8B, which co-localize with human NAT8 SNPs in NCBI dbSNP, are boxed. [file 1471-2350-9-25-S2.pdf]

Additional data file 2

File format: pdf

Title and description of the data:

Nucleotide sequence alignment between human *NAT8* gene and its duplicated pseudogene *NAT8B.*

The alignment includes the entire gene along with 541 bp of the 5´ upstream and 700 bp of 3´ downstream regions. The untranslated mRNA and the protein-coding regions are marked with thin and bold lines, respectively. Nucleotides typed in **bold** indicate SNPs detected by resequencing and their position relative to ATG. Divergent positions between *NAT8* and *NAT8B* overlapping with SNPs listed for the human *NAT8* gene in NCBI dbSNP (http://www.ncbi.nlm.nih.gov/projects/SNP/) are boxed.

Untranslated mRNA

Protein-coding regions

SNPs in dbSNP database that may represent PSVs

-1255 SNP positions detected by resequencing

PCR primer “NAT8 2kb F”

```
NAT8_29_10_06      CAGAGGAACTGCCCAGCCAAGCTCAGGCAAAATTGCTGACTTCAGACTCATGAACTAAAT 60
NAT8B_20_10_06     CAGAGGAACTGCCCAGCCAAGCTCAGGCAAAATTGCTGACTTCAGACTCATGAGCTAAAT 60
                   ***************************************************** ******

NAT8_29_10_06      AAATATTTGTTTCATTTGGTGGTTGTTCTTATGCAACATTATGCTGGCACTAGAAAAATG 120
NAT8B_20_10_06     AAATATTGATTTCATTTTATGATTGTTCTTATGCAACATTATCCTGGCACTAGAAAAATG 120
                   *******  ********  ** ******************** *****************
                                859
NAT8_29_10_06      AAACAGATGGGAATCAAAAAGACTGAATGAAGCAGAAACCCAGAAGCTACCCCACAAGCA 180
NAT8B_20_10_06     AAACAGATGGGAATCAAAAAGACTGAATGAAGCAGAAACCCAGAAGCTACCCCACAGGCA 180
                   ******************************************************** ***

NAT8_29_10_06      ATCACATGGGACTCAGATGTGAATGGACAATAAGCTTTTATTGCATTGAAAGGTCATTGC 240
NAT8 B20_10_06     ATCGCATGGGACTCAGATGTTAATGGACAATAAGCTTTTATTGCACAGAAAGGTCATTGC 240
                   *** **************** ************************  *************

NAT8_29_10_06      AGTGAAAGGTTGGGGATTGCTTGCTGCTACAGCTGAATGGATTCTATTCTGACCAATACA 300
NAT8B 20_10_06     AGTCAGAGGTTGGGGATTGCTTGCTGCTACAGCCGAATGGATTCTATTCTGACCAATACA 300
                   *** * *************************** **************************

NAT8_29_10_06      CACAGAAAGAGATCACAGACTCCCTACCTTAGAAGAAGGGAGGTGGTAGATGAAATGAAC 360
NAT8B 20_10_06     GAAGGAAAGAAATCATAGACGCCCTGCCTGAGCAGAAGGGAGGTGATAGATGAAATGAAC 360
                    *  ****** **** **** **** *** ** ************ **************

NAT8_29_10_06      TGTATGAAGAGCCACTAGCCTGGCCCACACACAGAAGAAGGACTGGCCCGTCTTCTTGAA 420
NAT8B 20_10_06     TGTATGAAGATCCACCAGCCTGGCCCACACGTGGAAGAAGGACTGGCCCGTCTTCTTGAA 420
                   ********** **** **************   ***************************

NAT8_29_10_06      GCCCATGCTCTGGTAGAGGGCCATAGCAGAGAGCTGGATGGTGCCGGTGTCCAGGATAAC 480
NAT8B 20_10_06     GCCCAAGCTCTGGTAGAGGCCCATGGCAGAGAGCTGGATGTTGCTGGTGTCCAGGACAAC 480
                   ***** ************* **** *************** *** *********** ***

NAT8_29_10_06      TTCACTGTAGCCCTGGTCCCGGGCAAACTGGAGGACAGTCCTGACCAGGGCTTTTGCTAT 540
NAT8B 20_10_06     TTCACTGTAGCCCTAGTCCCGGGCAAACTGGAGGACAGTCCTGACCAGGGCTTTTGCTAT 540
                   ************** *********************************************
                                             428
NAT8_29_10_06      CCCCTGACGACGGTGCTCACTGTCCACAAAGAGATGAAACAGCTGCAACCGCTTCTCCCT 600
NAT8B_20_10_06     CCCCTGACCACGGTGCTCATTGTCCACAGAGAGATGAAACAGCTGCAACCGCTTCTCCCT 600
                   ******** ********** ******** *******************************
```

```
                        390                        361
NAT8_29_10_06     CAAGGTGGGATCATCAACAGGCAGAGCTCCTACCATGCCCACCACCTTCTCTTCAGACTC 660
NAT8B_20_10_06    CAAGGTGGGATCATCAACGGGCAGAGCTCCTACTGTGCCCACCACCTTCTCTTCAGATTC 660
                  ****************** **************  ********************** **

NAT8_29_10_06     AGCCACCCAGAAGCAGGAGCCACGCTCACTCAGGTAGGATTTGGTAATGTCAGACATGTC 720
NAT8B_20_10_06    ACCCACCCAGAAGCAGGAGCCACACTCACTCAGGTAGGATTTGGTGATGTCAGACATGTC 720
                  * ********************* ********************* **************
                                                                          218
NAT8_29_10_06     TGTGCACAATGTCATGTCCACATACTCCGTCCAGGGTTTTTGGCAAGGAACCACAGGGC 780
NAT8B_20_10_06    TGTGCGCAATGCTATGTCTACATACCGCGTCCAGGGTTTTTGGCAAGGAACCACAGGGC 780
                  ***** *****  ***** ******  *********************************

NAT8_29_10_06     AGGGAAGAGGCTGATGCTGAACACGAGGGCTAGAAGCCAGGATCCAGAGACCAGGAGTAG 840
NAT8B_20_10_06    AGGAAGGAGGCTGAGGCTGAACACGAGGGCCAGAATCCAGGAGCCAGAGACCAGGAGTAG 840
                  *** * ******** *************** **** ****** *****************

NAT8_29_10_06     GGCGAGGGGCCCCCCAAGTAAGAGTATGAGGGTTCGAGGCAGCTTCAGCAATTGCCGGAA 900
NAT8B_20_10_06    GGCAAGGGCCCCCCCAAGTAAGAGTATGAGGGTTCGAGGCAGCTTCAGTAATCGCCGGAA 900
                  *** **** *************************************** *** *******
                                                                        41
NAT8_29_10_06     GGTGGCTGGGGCATGCTCGGCCATCCCCCGGGAGAGCAAGCCCACAACCCACTGGCGGTC 960
NAT8B_20_10_06    GGTGGCTGGGGCGTGTTCGGCCATCCCCCGGGAGAGCAAGCCCACGACCTACTTGCGGTC 960
                  ************ ** ***************************** *** *** ******

NAT8_29_10_06     GCTCTCCTGGTATTTGCGGATGTGACAAGGAGCCATGGACAGACTTCTGTGTCTGAAATC 1020
NAT8B_20_10_06    GCTCTCCTGGTATTTGCGGATGTGATAAGGAGCCATGGACAGACTTCTGTGTCTGAAGTC 1020
                  ************************* ******************************* **

NAT8_29_10_06     TCTAAGTCCAGGAGACAGAGCTAGGCCTCCCTGCACGCCTTTACGTCAGAC-TGGGGAAG 1079
NAT8B_20_10_06    ACTGAGTCCAGGAAACAGAGCTAGGCCTCCCTGCACGCCTTTACGCCAGGCCTGGGGAAG 1080
                   ** ********* ******************************* *** * ********

NAT8_29_10_06     AGAGAGAAAATCATGTGAGTGCCTGCATGGCTCCCAGCCTTGCAGGAACAGGGAGACCTG 1139
NAT8B_20_10_06    AGAGAGGAAACCACGTGAGTGCCTGCATGGCTCCCAGCCTTGCAGGAACAGGGAGACCTG 1140
                  ****** *** ** **********************************************

NAT8_29_10_06     CTCAAGGGTGTGTCTTTCCGCGTTTGCCCATGAGGAAGCAGGCCTCTGCCACTGGGAGAA 1199
NAT8B_20_10_06    CTTAAGGGTGTGTCTTTCCGCGTTTGCCCATGAGGAAGCAGGCCTCTGCCACTGGGAGAA 1200
                  ** *********************************************************
                                -222
NAT8_29_10_06     GGTAGGGTCAGAAAGACCTGCATTGGAATCTGGTTCCATCCCTTTCTAGCTGTGTGACCT 1259
NAT8B_20_10_06    GGTAGCATGAGAAAGACCTGCATTGGAATCTGGCTCCATGCCTTTCTAGCTGTGTGACCT 1260
                  *****  * ************************ ***** ********************

NAT8_29_10_06     TAGGCATGTCACTTAACCTCTCTGAGCCTCCATTTCTTCAACTATAGAACAGGGGAGAAG 1319
NAT8B_20_10_06    TGGGCATGTCACTTAACCTCTCTGAGCCTCCACTTCTTCATCTATAGAACAGGGGAGAAG 1320
                  * ****************************** ******* *******************
                                                        -365
NAT8_29_10_06     AAAACCTGCCTTCTAGAGTTGTTTTGAAGATTGAATAAGATGCCTTCTATAAAGCCCTGG 1379
NAT8B_20_10_06    AAAACCTGCCTTCTAGAGTTGTTTCGAAGATTGAATAAAATACCTTCTATAAAGCCCTGA 1380
                  ************************ ************* ** *****************

NAT8_29_10_06     TAGATGAACTACCCACCGCTCTTCCTTTTTCTGTTTCCTTTTTCTGGCTCTCTCAGCTTC 1439
NAT8B_20_10_06    TAGATGAACTACCCACCACTCTTCC---CTCTGTTTCCTTTTTCTGGCTCTCTCAGCTTC 1437
                  ***************** *******    *******************************
```

```
NAT8_29_10_06      TCCGTGCATTGAGGGGGCACCTACCTCAGGCAGGCCACACGTGCAACCCTTTTGTCCTTC 1499
NAT8B_20_10_06     TTGGTGCATTGAGAGAGTACCTACCTCAGGCAGGCCACATGCCCAACCCTTTGGTGCTTC 1497
                   *  ********** * * ********************* *  ********* ** ****

NAT8_29_10_06      TCCAGGGACCATACATCGCAAAAATTCTCTGTCCAA-CACAAGTTTCAGGAAAGGCTCCC 1558
NAT8B_20_10_06     TCCAGGGACCATACATCGCAGAAATCCTGTGACCAAGCACAAGTTTCAGGAAAGGCTCCC 1557
                   ******************** **** ** ** **** ***********************

NAT8_29_10_06      TCCAGCTTTATTGTAAGTTTTTAGGTTGCTGAAGTGGCATAAGAGCCAAGCATAAAAAAC 1618
NAT8B_20_10_06     TCCAGCTTTATTGTAAGTTTTTAGGTTGCTGAGGTGGCATAAGTGCCAAGCATAAAAAAC 1617
                   ******************************** ********** ****************

NAT8_29_10_06      AGCCCAGCCTCCAGATCCCAAGGAAGGTGCATGCTCAGAGCACTGTCCTTCCACCCAGTC 1678
NAT8B_20_10_06     AGCCCAGCCTCCAGATCCCAAGGAAGGTGCATGCTCAGAGCACTGTCCTTCCACCCAGTC 1677
                   ************************************************************

NAT8_29_10_06      CCCTCACCTGTCACCACAAGAGCCTTGAGTGTCCAGGGAGCTTCAGCTCTCAGCCGCTCA 1738
NAT8B_20_10_06     CCCTCACCTGTCACCACAAGAGCCTTGAGTGTCCAGGGAGCTTCAGCTGTCAGCCGCTCA 1737
                   ************************************************ ***********

                                     -763
NAT8_29_10_06      CTGGCATCCAGGAGATACCGTCTGGGGCTTGCTCACCCCTGGAAAGTAGACTGCCTGCCT 1798
NAT8B_20_10_06     CTGGCATCCAGGAGATACCGTCTGGGGCTTGCTCACCCCTGGAAAGCAGGCTGCCTGCCT 1797
                   ********************************************** ** **********
                 -803
NAT8_29_10_06      TATTGCTTGGCTCCTGAATGTTTGGTAATCATCAACCCTAGGGTCAAAGAGCTGGGCTGT 1858
NAT8B_20_10_06     CATTGCTTGGCTCCTGAATGTTTGGTAATCATCAACCCTAGGGTCAAAGAGCTGGGCTGT 1857
                    ***********************************************************
                                                                 -911
NAT8_29_10_06      AACACAGCAGGGAGCCCAGTGTGCATTCCTCTTTGTGGTCAAAGGGAACGACCTGGAGGG 1918
NAT8B_20_10_06     AACACAGCAGGGAGCCCAGTGTGCATTCCCCTTTATAGTCAAAGGGAATGGCCTGGAGGG 1917
                   ***************************** **** * *********** * *********

NAT8_29_10_06      GGAGACTCAGGGTCTGGAACCGCCTGGGTTCCTTGCCCTTCTGGAAGGCCTGCCACCTCT 1978
NAT8B_20_10_06     GGAAACTCAGGGTCTGGAACCGCCTGGGTTCCTTGCCCTTCTGGAAGGCCTGCCACCTCT 1977
                   *** ********************************************************
                                                                         -1041
NAT8_29_10_06      CTTGCCTGTTCAGGCCCCGTGCATCTCTCTGCCACCTGGCACACTTAGTACTCTCGGTCC 2038
NAT8B_20_10_06     CTTGCCTGTTCAGGCCCTGGGCATCTTTCTGCCACCTGGCACACTTGATACTCTC----- 2032
                   ***************** * ****** *******************  *******

NAT8_29_10_06      CTGTCCACATATACGATCCACCCTGGTCCTTGTACACATATACGACGAGCCTGAGAACTG 2098
NAT8B_20_10_06     -----------TGAGATCCACCTTGGTCCCTGTACACATATACCACGAGCCTGAGAACTG 2081
                              *  ******** ****** ************* ****************
                                                              -1152/-1153
NAT8_29_10_06      TACCATTCACCCTGCAATCAGTCACCATTGGCCAGAAGCCGAGTCCCCATGCTTGGCAAC 2158
NAT8B_20_10_06     TGCCATTCACCCTGCACTCAGTCACCATTGGCCAGAAGCCGAGTCCCCACACTTGGCAAC 2141
                   * ************** ********************************  *********

NAT8_29_10_06      AAGGCCCTTCCCAGCTAGGCTTCTCCTCGCTGAGGTTTCCCTGGTCCACGAAGCATCTGT 2218
NAT8B_20_10_06     AAGGCCCTTCCCAGCTAGGCTTCTCCATGCTGAGGTTTCCCTGGTCCACAAAGCATCGAT 2201
                   **************************  ********************* *******  *
```

```
                                                   -1255
NAT8_29_10_06      TGTTGGCAGTTCCTTGCACTCACCTCACCAACCCCCATCTCCACCCATTCATTGATTCAG 2278
NAT8B_20_10_06     TGTTGGCAGTTCTTTGCACTCACCTCACCAACCCCCACCTCCACCCATTCATTGATTCAG 2261
                   ************ ************************ **********************

NAT8_29_10_06      TCAACAGTTGTGTGTCATGCACTTCCTGTGTCCTGGGGTCATTTCAGCCATGAGCAAGGT 2338
NAT8B_20_10_06     TCAACAGGTGTGTGCGATGCACTTCCCATGTCTGGGGGTCATTTCAGCCATGAGCAAGGT 2321
                   ******* ******  **********  ****  PCR primer “NAT8 2kb R”

NAT8_29_10_06      TGACATTCGGCTTGACAGGCATGGCTGTGTGTGGCTTGTGTGTGTTCTGGCTGTTGGTCA 2398
NAT8B_20_10_06     TGACATTCGGCT-GACAGGCATGGCTGTGTGTGGCTTGTGTGTGTTCTGGCTGTTGGTCA 2380
                   ************ ***********************************************


NAT8_29_10_06      GAGGCTGTGCCACACCCGTTGTTAAATATTTTGCACTGCAGATGTTGGGGAGGCCCAAAT 2458
NAT8B_20_10_06     GAGGCTGTGCCACACCCGTTGTTAAATATTTTGCACTGCAGATGTTGGAGAGGCCCAAAT 2440
                   ************************************************ ***********

NAT8_29_10_06      TCATGCTCTT
NAT8B_20_10_06     TCAAGCTCTT
                   *** ******
```
